# The psychology of incels: a dual-pathways framework for mental health, ideology, and harm

**DOI:** 10.3389/fpsyt.2026.1936687

**Published:** 2026-09-23

**Authors:** Changiz Mohiyeddini

**Affiliations:** 1 Michael Garron Hospital, Toronto East Health Network, Toronto, ON, Canada; 2 Department of Family and Community Medicine, Temerty Faculty of Medicine, University of Toronto, Toronto, ON, Canada

**Keywords:** black pill, incels, involuntary celibacy, loneliness, misogyny, psychopathology, self-harm, violence risk

## Abstract

The incel, or *involuntary celibate*, phenomenon refers to an online subculture in which predominantly heterosexual men interpret unwanted romantic or sexual non-partnership through narratives of rejection, biological determinism, grievance, and gendered injustice. This Hypothesis and Theory article argues that incel psychology is usefully organized along two interacting dimensions of vulnerability, adapting and broadening a recently proposed dual-pathways model of incel harm. The first dimension is experiential vulnerability: loneliness, social exclusion, bullying, low perceived mate value, depression, anxiety, rejection sensitivity, and poor social support. The second is ideological–dispositional risk: misogynistic grievance, entitlement, dehumanization, narcissistic vulnerability, and fatalistic “black-pill” beliefs. Whereas the original model was developed to predict harmful attitudes and beliefs, the present framework uses experiential vulnerability and ideological–dispositional risk more broadly to organize incel identification and to specify differential associations with self- and other-directed harm. Online networking is proposed here as a distinct amplifying element. The dimensions may interact, though cross-sectional evidence cannot establish direction. The framework generates three provisional hypotheses concerning differential associations with self-directed harm, other-directed harmful attitudes, and the hypothesized amplifying role of online networking. The article distinguishes involuntary celibacy as a condition, incel identity as a self-label, and incel ideology as a misogynistic, fatalistic worldview. Many self-identified incels experience significant distress and may be at elevated risk for self-directed harm. A subgroup may present elevated outward-directed risk when distress combines with violent ideation, dehumanization, revenge fixation, and online reinforcement. Claims here concern male, heterosexual incel communities, which dominate the empirical literature; female incel (“femcel”) communities remain under-researched and are not the focus of this article.

## The psychology of incels: a dual-pathways framework

The term *incel* is a portmanteau of *involuntary celibate*. In contemporary research and public discourse, however, it refers not simply to unwanted sexual inactivity but to a subcultural identity and, in some cases, an ideological worldview. Self-identified incels commonly describe themselves as unable to obtain sexual or romantic relationships despite desiring them. In more ideological incel spaces, this experience is interpreted through fatalistic narratives of biological hierarchy, female sexual selection, feminism, and male dispossession (1, 2).

This article argues that incel psychology is best understood through an interaction between experiential vulnerability and ideological–dispositional risk. The experiential dimension includes loneliness, social exclusion, rejection, bullying, poor mental health, low self-esteem, low perceived mate value, and perceived lack of social support. The ideological–dispositional dimension includes misogynistic grievance, entitlement, outgroup hostility, hostile sexism, dehumanization, and fatalistic “black-pill” beliefs. This framing helps avoid two common errors: reducing incels to mentally ill lonely men, or reducing all incel identification to violent extremism.

This framework adapts the dual-pathways hypothesis of incel harm advanced by Costello, Whittaker, and Thomas (3), whose study of 561 self-identified incels found that poor mental health and ideological adherence were each roughly twice as predictive of harmful attitudes and beliefs as online social networking. The present article departs from that model in three respects. First, their model is specifically a model of *harm*, a set of predictors of harmful attitudes and beliefs, whereas the present article uses the same two dimensions more broadly, both to describe routes into incel identification and to organize differential associations with self- and other-directed harm. Second, the distinction is made explicit throughout: *pathway* or *dimension* denotes a dimension of risk rather than a demonstrated developmental sequence, and the role of each dimension in entry into incel identification is separated from its association with harmful outcomes. Third, their account situates the two pathways within a needs, networks, and narratives (3N) framework; the present article foregrounds need and narrative as the two dimensions while retaining networking as a distinct, amplifying element rather than collapsing it into their interaction. **Figure 1** summarizes the adapted dual-pathways framework used in this article. The figure represents experiential vulnerability and ideological–dispositional risk as interacting dimensions, with online networking functioning as an amplifying element rather than as a separate developmental pathway. The framework’s principal contribution is therefore not the identification of entirely new risk factors, but their integration into a differentiated psychological account that separates condition, identity, and ideology; distinguishes self-directed from other-directed harm; and translates these distinctions into clinical, educational, and research implications. Three hypotheses follow from this framework and are stated formally below (Hypotheses 1–3): experiential vulnerability should show a comparatively stronger association with self-directed harm; ideological–dispositional risk should show a comparatively stronger association with other-directed harmful attitudes; and grievance-reinforcing online networking should amplify either association. The sections that follow assemble the evidence bearing on each. In short, the source model predicts who holds harmful attitudes; the present framework additionally specifies who is drawn to incel identification, which harms are more likely for whom, and how clinicians and educators might respond, and it renders these claims disconfirmable through the hypotheses stated below. A recent critical narrative review of the incel phenomenon and its public-health implications (4) offers a valuable synthesis of this literature; the present article differs in aim, providing not a survey of findings but an organizing, dimensional framework that separates condition, identity, and ideology and yields explicitly disconfirmable hypotheses.

**Figure 1:**
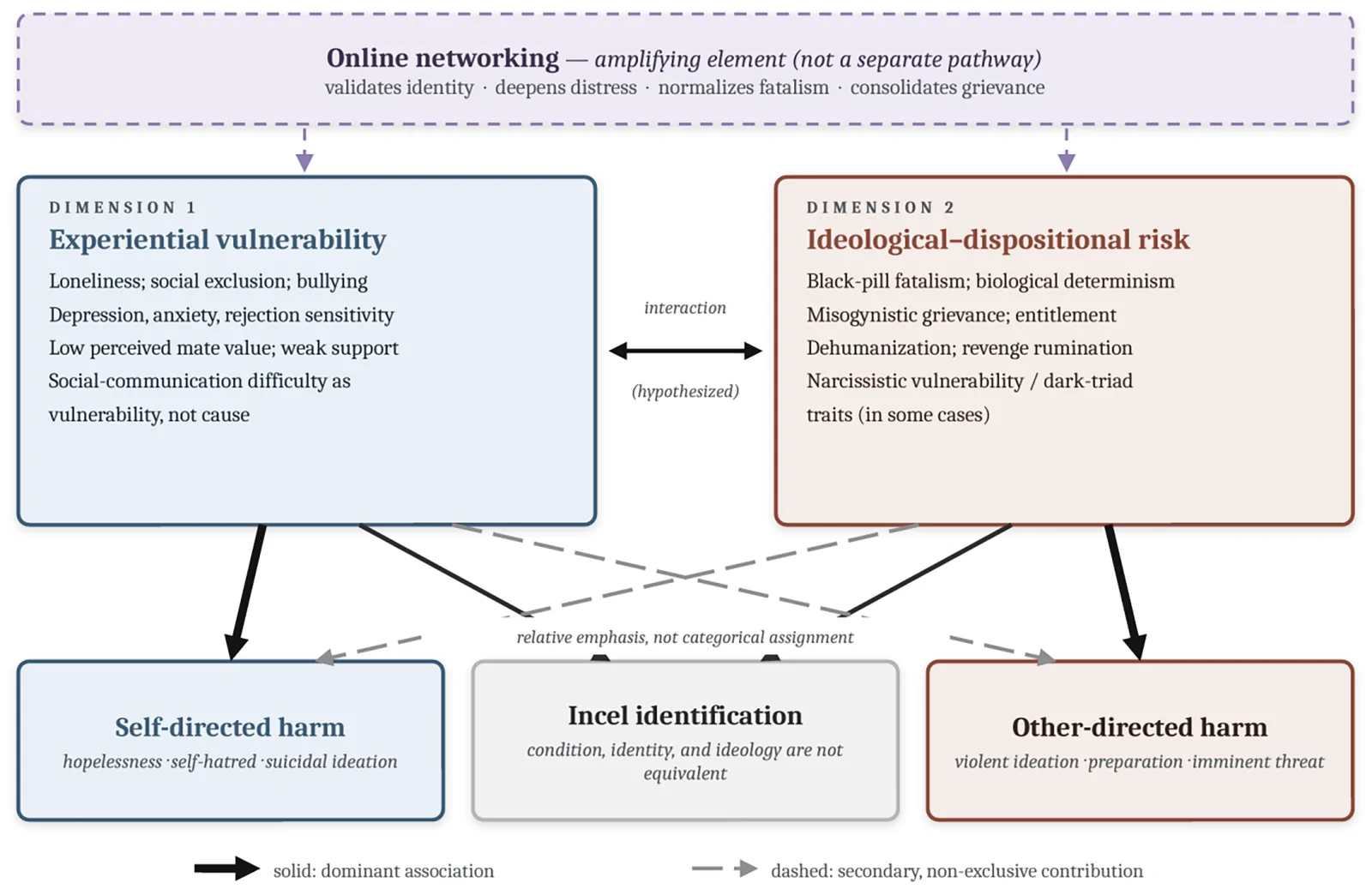
Conceptual dual-pathways framework for incel identification and harm. Experiential vulnerability refers to loneliness, rejection, poor mental health, low perceived mate value, and weak social support. Ideological–dispositional risk refers to black-pill fatalism, misogynistic grievance, entitlement, dehumanization, and revenge-oriented cognition. Online networking is represented as an amplifying element rather than a separate developmental pathway. The model distinguishes incel identification, self-directed harm, and other-directed harm as related but non-equivalent outcomes. Solid arrows denote the dominant association of each dimension; both dimensions contribute to all outcomes. Arrows indicate hypothesized associations rather than established longitudinal causal pathways. No relative difference in the strength of the two dimensions’ associations with incel identification is proposed.

The framing adopted throughout is dimensional rather than categorical, and in this respect it is aligned with contemporary dimensional approaches to psychopathology, most prominently the Hierarchical Taxonomy of Psychopathology (5). Experiential vulnerability comprises features (loneliness, depression, anxiety, rejection sensitivity, low self-esteem) that correspond broadly to the internalizing spectrum, whereas ideological–dispositional risk comprises features (entitlement, hostile attribution, dehumanization, narcissistic vulnerability, dark-triad traits) that resemble antagonistic externalizing characteristics. This alignment is offered as a heuristic orientation rather than a measurement claim: incel identification is not a diagnostic entity, and neither dimension has been formally located within dimensional nosologies. This correspondence clarifies the article’s central claim: incel-related distress and risk are better understood as continuous, transdiagnostic phenomena than as markers of a categorical disorder, and assessment should be differentiated accordingly.

These claims also connect to, and attempt to reconcile, two strands of prior work that have often been in tension. One strand, prominent in security and radicalization scholarship, foregrounds incel ideology as a driver of misogynistic violence (6); another, emerging from clinical and survey research, emphasizes the community’s profound distress and elevated suicidality and warns against over-securitization (3, 7, 8). The present framework treats these not as competing accounts but as descriptions of different pathways and outcomes within a heterogeneous population: experiential vulnerability speaks to the mental-health burden the first strand tends to neglect, ideological–dispositional risk to the harm the second foregrounds, and their interaction to why both literatures capture something real. Practically, this favors a public-health rather than a purely forensic posture, treating mental-health support and misogyny-focused prevention as complementary rather than alternative responses, while retaining proportionate threat assessment for the subgroup in which risk indicators are present.

A further distinction is necessary. Involuntary celibacy as a subjective condition is not equivalent to incel identity, and incel identity is not always equivalent to incel ideology. Many people are single, sexually inexperienced, socially anxious, or lonely without adopting misogynistic beliefs. Conversely, incel ideology involves a more structured interpretation of sexual and romantic exclusion as the result of an unjust, biologically determined, female-controlled sexual hierarchy (6). This distinction matters clinically, educationally, and scientifically because the intervention needed for loneliness differs from the intervention needed for violent misogynistic grievance.

## Scope and evidence base

This article offers a narrative conceptual synthesis of recent empirical, theoretical, and applied literature on incels, rather than a systematic review or meta-analysis, with particular attention to psychological distress, social isolation, perceived mate value, identity formation, black-pill fatalism, misogyny, and harm. A recent systematic scoping review provides a useful map of this rapidly growing field (1). Policy guidance, including UK government material on incels and safeguarding (9), is used here for applied framing rather than as primary psychological evidence. Throughout, claims based directly on empirical studies are distinguished from theoretical synthesis and applied implications.

Literature was identified through targeted searches of PsycINFO, PubMed, Scopus, and Google Scholar using combinations of terms relating to incels, involuntary celibacy, incel ideology, mental health, loneliness, misogyny, radicalization, self-harm, and violence. Priority was given to peer-reviewed empirical studies, systematic or scoping reviews, and clinically or theoretically relevant analyses available by July 2026. Reference lists of key reviews and primary studies were also examined. Because the literature remains methodologically heterogeneous and relatively small, studies were synthesized conceptually rather than subjected to formal quality scoring or meta-analysis, and policy documents were used only for applied safeguarding context. Search terms combined population descriptors (incel, involuntary celibate, “black pill”) with content descriptors (mental health, loneliness, depression, anxiety, misogyny, radicalization, self-harm, violence, perceived mate value, and identity). Inclusion prioritized peer-reviewed primary studies and reviews reporting psychological, clinical, or ideological data on self-identified incels; excluded were non-scholarly commentary and sources without empirical or theoretical bearing on the framework, apart from government safeguarding guidance retained for applied context. The two dimensions were derived initially from the source dual-pathways model and refined against the retrieved evidence, and studies were then organized under the dimension or dimensions to which they most directly spoke. Because primary samples are typically forum-recruited, self-selected, and cross-sectional, prevalence and causal inferences are treated throughout as provisional. Searches were run through July 2026. A representative string was (“incel” OR “involuntary celibate” OR “black pill”) AND (“mental health” OR loneliness OR depression OR misogyny OR radicali* OR “self-harm” OR violence). Titles and abstracts of retrieved records were screened for relevance to the psychology, mental health, ideology, or harm of self-identified incels; full texts of retained records were read; and reference lists of key sources were hand-searched. Consistent with the Hypothesis and Theory article type, which advances and organizes a theoretical model rather than exhaustively cataloguing a literature, no formal quality-appraisal or risk-of-bias instrument was applied; instead, the design and strength of each cited study are noted in text where they bear on a claim.

The incel literature remains methodologically young. Many studies rely on self-selected samples, forum data, cross-sectional designs, or surveys of people willing to identify with a stigmatized online label (1). Forum studies may overrepresent the most extreme participants, whereas surveys may miss hidden, ambivalent, or disengaged individuals. The conclusions below should therefore be read as evidence-informed rather than conclusive, and quantitative figures cited throughout should be interpreted against these sampling constraints. Throughout, causal language describes hypothesized pathways only; the available evidence is predominantly cross-sectional and cannot establish temporal or causal relationships, and hypothesized links are signaled as such.

Two demographic correctives bear on the article as a whole. Although incels are frequently portrayed as predominantly white and right-wing, more recent samples describe ethnically diverse communities (10, 11), and the largest primary study to date reports both ethnic and political diversity, with substantial proportions identifying as centrist or left-leaning rather than right-wing (3; see also 8). These findings caution against equating incel identification with organized far-right extremism. The most consistently documented elements of incel ideology are misogynistic grievance and black-pill fatalism; their alignment with broader political extremism is more variable and contested.

The Costello, Whittaker, and Thomas study provides the principal empirical foundation for the adapted framework because of its comparatively large sample and direct assessment of mental health, ideology, networking, and harmful attitudes. Its forum-recruited, cross-sectional, self-report design nevertheless limits causal interpretation and population-level generalization.

## Conceptual distinctions: condition, identity, and ideology

A psychologically precise account should distinguish three levels.

First, involuntary celibacy as a *condition* refers to the unwanted absence of sexual or romantic relationships. This condition may be associated with loneliness, social anxiety, depression, shame, neurodevelopmental difficulties, limited dating experience, or poor perceived attractiveness (12, 13). However, the condition itself does not imply misogyny or extremism.

Second, incel *identity* refers to the adoption of “incel” as a self-label. This identity may provide explanation and belonging, and it may help individuals name an otherwise private and humiliating experience (14). However, identity adoption may also consolidate hopelessness if the label becomes totalizing.

Third, incel *ideology* refers to a worldview in which romantic failure is explained through deterministic and misogynistic beliefs. This ideology often emphasizes immutable physical traits, female hypergamy, feminism, sexual-market hierarchy, resentment toward sexually successful men, and the belief that ordinary self-improvement is futile (2, 15). The most consequential ideological form is the “black pill,” which frames inceldom as biologically fixed and socially inescapable.

These levels can overlap, but they are not interchangeable. A socially isolated young man who feels unwanted may require psychological support and relational skill-building. A person who dehumanizes women, glorifies attackers, or expresses revenge fantasies requires a more intensive risk-assessment response (7). These individual and group-level processes also operate within broader cultural scripts that link masculine worth to sexual success, status, dominance, and female validation; incel ideology radicalizes these scripts rather than creating them ex nihilo.

## Dimension 1: experiential vulnerability

The first dimension of the framework is experiential vulnerability; the evidence assembled in this section grounds Hypothesis 1. Empirical studies report elevated loneliness, depression, anxiety, poor social support, rejection sensitivity, low self-esteem, and lower relational well-being among self-identified incels relative to non-incel men (12, 13, 16). Survey work has found high rates of self-reported difficulty: in one sample, roughly 95% reported depression and 94% reported anxiety, with about 37% reporting a formal diagnosis of depression (and a comparable proportion a formal diagnosis of anxiety) (8), and in the largest primary study to date (N = 561) approximately one in five reported daily suicidal thoughts (3).

Functional and social impairment accompany this distress. In the same large study, participants reported very high levels of loneliness, about 18% were not in education, employment, or training, and roughly half still lived with parents (3). These figures should be read against the sampling limitations noted above: such samples are self-selected and often forum-recruited, and may overrepresent the most distressed or most engaged individuals. They nonetheless support the view that incel identification is frequently associated with substantial distress and cannot be understood through ideology alone.

Loneliness is central. It should not be reduced to objective isolation. Loneliness refers to the perceived gap between desired and actual social connection. An individual may interact frequently online and still feel profoundly lonely if those interactions do not provide intimacy, trust, recognition, or reciprocal care (13). Incel forums may temporarily reduce loneliness by providing group belonging, but they may also reinforce the belief that members are permanently excluded from ordinary intimacy; this is a theoretical inference rather than a directly tested finding.

Low perceived mate value is another important experiential factor. Incel discourse often centers on beliefs about height, facial structure, attractiveness, race, hairline, body composition, and other physical characteristics. Consistent with this, self-identified incels report lower self-perceived mate value and a more external locus of control over their singlehood than non-incel single men (17). These beliefs can become rigid and global: the individual does not merely think, “I have been rejected,” but “I am biologically excluded from relationships.” Such beliefs may increase avoidance, reduce attempts at social engagement, and intensify body-image rumination, though this developmental sequence is a clinical inference not yet directly tested in this population.

Depression and anxiety may be associated with stronger incel identification or with processes that maintain it. Depression is commonly associated with hopelessness, negative self-evaluation, and reduced expectation of reward; a foreshortened and negative temporal perspective has been observed in this population (12). Social anxiety can promote avoidance of interpersonal situations and prevent corrective experiences. If an individual avoids social contact because rejection feels inevitable, the absence of positive experience then appears to confirm the original belief.

Some research also discusses autism-spectrum traits or social-communication difficulties in relation to incel identification. In the largest primary sample, roughly one-third of participants scored above a threshold warranting autism referral (3), and clinical reviews note elevated neurodevelopmental difficulty in this population (7). This association must be handled carefully. Neurodivergence does not cause misogyny, entitlement, or violence. The more defensible interpretation is that social-communication difficulties, bullying, isolation, or chronic rejection may increase vulnerability to online spaces that offer explanation and belonging (7). Where present, such difficulties may also shape how rejection and ambiguous dating cues are interpreted and may heighten the appeal of structured, rule-based explanatory systems; these possibilities are hypotheses to be tested, not established mechanisms.

### Existential isolation and identity appeal

Work on existential isolation is useful for understanding why the incel label can become psychologically compelling. Interpersonal loneliness concerns lack of connection; existential isolation concerns the belief that others cannot understand one’s subjective experience. For some incel-identified individuals, the central wound may not only be “I am alone,” but “No one understands what it is like to be me.” Papandreou et al. (14) characterize the resulting identity trajectory as one of “rot, rage and blame.”

Incel communities may respond directly to existential isolation by offering a vocabulary, a narrative, and a peer group. Terms such as “black pill” (the fatalistic worldview), “Chad” and “Stacy” (stereotypically attractive, sexually successful men and women), “normie” (someone perceived as socially conventional and romantically integrated), and “looksmaxxing” (efforts to enhance appearance) do not merely describe social categories; they organize experience. They provide an interpretive system through which personal rejection becomes intelligible (15). This may be psychologically powerful because it converts private shame into shared identity.

However, the same process may also deepen distress, cognitive rigidity, and ideological identification. The community may validate pain while supplying distorted explanations. The person receives recognition, but the recognition may be conditional on accepting fatalistic and misogynistic assumptions (14). In this way, online belonging can reduce immediate isolation while maintaining the worldview that produces long-term hopelessness.

## Dimension 2: ideological and dispositional risk

The second dimension of the framework is ideological–dispositional risk, and the evidence assembled here grounds Hypothesis 2. It is defined not primarily by loneliness, but by the interpretation of loneliness through grievance, entitlement, misogyny, and dehumanization (6, 15). It is through this dimension that incel ideology may become socially harmful.

The black-pill worldview is a central ideological mechanism. It asserts that romantic and sexual success are determined overwhelmingly by immutable biological traits (2). The term extends the manosphere’s “red pill”/”blue pill” metaphor, borrowed from the film The Matrix, in which taking the red pill signifies awakening to a hidden truth. In manosphere discourse, the red pill claims to expose a sexual marketplace favoring women; the black pill adds biological determinism and fatalism (18). Within this worldview, ordinary agency is rejected. Therapy, social learning, education, exercise, improved communication, or expanded social networks may be dismissed as futile. This creates an anti-therapeutic logic: the ideology explains pain by denying the possibility of meaningful change, and seeking mental-health support is actively discouraged within parts of the community (7).

Misogyny intensifies this fatalism. Some incel discourse portrays women as shallow, cruel, manipulative, hypergamous, or collectively responsible for male suffering (6). Women’s sexual autonomy is reframed as male victimization. This is psychologically important because it converts personal disappointment into moral grievance. For some individuals, rejection may then be experienced not only as sadness or shame but also as injustice (15).

Entitlement is therefore a key construct. Not all lonely or sexually inexperienced men are entitled. Entitlement emerges when sexual or romantic access is perceived as something owed. When that perceived entitlement is denied, anger may be directed toward women, sexually successful men, feminism, or society (6). This structure resembles broader grievance-based ideologies in which perceived humiliation is externalized into outgroup blame (15).

Dispositional features have also been examined. Pathological narcissism has been proposed as one framework for some incel dynamics, particularly aggrieved entitlement, humiliation, envy, hypersensitivity to rejection, and oscillation between self-loathing and superiority (19). Relatedly, dark-triad traits have been examined in several studies; the largest primary study includes the Dark Triad among its constructs and reports elevated angry rumination and revenge planning alongside heightened rejection sensitivity (3). These observations should not be overextended. The literature does not justify diagnosing incels as narcissistic, or as dark-triad-elevated, as a group, and the relevant effect sizes are modest and sample-dependent. They support cautious attention to narcissistic vulnerability and grandiosity as features in some individuals, not a categorical trait claim.

### Cognitive-discursive patterns in incel ideology

It is tempting to describe the regularities of incel ideology as “cognitive distortions.” A more precise formulation is “cognitive-discursive patterns,” because not all of these patterns have been empirically established as individual-level cognitive traits as opposed to features of online discourse.

One pattern is a distorted perception of societal attitudes. Incels appear to overestimate how much society blames or despises them and to underestimate how much others sympathize with them (20; see also 21). Because this perception gap rests on a measurable misappraisal rather than an accurate reading of social reality, it is a potential point of intervention, a theme addressed below.

A second pattern is the overemphasis on physical attractiveness and status in mate selection. In the first formal study of incel mating psychology, self-identified incels overestimated the importance of physical attractiveness and financial prospects to women and underestimated traits such as warmth, kindness, humor, and intelligence (17). Two qualifications matter. First, the same study found that non-incel single men also overestimated the importance of attractiveness and financial prospects, so this misperception is not unique to incels and is, at most, a difference of degree. Second, and contrary to the assumption that incels hold unrealistically high standards, incels in that study reported *lower* minimum mate-preference standards than non-incel single men (17). The black-pill conclusion that looks are deterministic therefore reflects an interpretive frame more than an accurate reading of mate preferences.

A third pattern is hostile attribution: in incel discourse, ambiguous social events are often interpreted as contempt, rejection, or evidence of female cruelty. Stronger empirical evidence is needed, however, before hostile attribution can be described as a reliably measured cognitive trait among incels rather than a discourse regularity.

A fourth pattern is confirmation bias. Incel forums often treat rejection experiences as proof of black-pill claims while dismissing counterexamples (2). Again, this is well supported as a discourse pattern, but more research is needed to establish its status as a measured cognitive process specific to incels. Together, these patterns suggest that incel ideology may be sustained through misperception, selective attention, and confirmation bias, although the status of these processes as individual-level cognitive mechanisms remains to be established.

This distinction matters. Scientific writing should avoid treating interpretive patterns observed in online discourse as established clinical mechanisms unless empirical evidence supports that move.

## Interaction between the two dimensions, and the role of networking

The experiential and ideological dimensions may reinforce one another, though the evidence for this reciprocal process is predominantly cross-sectional and its direction has not been established. A person experiencing loneliness, depression, bullying, or low perceived mate value may search for explanation. Incel ideology offers one: the problem is not contingent, modifiable, or interpersonal, but biological and structural. This explanation may reduce uncertainty while increasing hopelessness. “Interaction” is used here in a conceptual rather than statistical sense, denoting potential reciprocal reinforcement; the available literature does not establish whether the joint effects are additive, multiplicative, mediated, or moderated.

Conversely, ideological immersion may worsen psychological distress. Black-pill content may intensify depression by portraying the future as closed. Misogynistic forums may reduce opportunities for genuine relationships by increasing contempt toward women. Dehumanizing language may erode empathy. Group norms may punish self-improvement by labeling it delusional or traitorous. On this account the ideology may maintain the very isolation it claims to explain. This cycle is best framed as a plausible hypothesis rather than a demonstrated developmental sequence; longitudinal designs are needed to test it, and at least some scholarship argues that ideological and suicidal content may be downstream of community indoctrination rather than a gateway into it (7).

The model adapted here is, in its original form, an account of needs, networks, and narratives (the 3N framework) in which online social networking is a distinct contributor alongside psychological need and ideological narrative (3). In the source study, networking was modeled as an independent predictor of harmful attitudes rather than as a moderator; the present framework goes beyond that model in proposing that its influence may be better understood as amplifying either dimension, a conjecture formalized below as Hypothesis 3 rather than an established finding. Networking is empirically separable: in the source study it independently predicted harmful attitudes, albeit less strongly than mental health or ideology, and greater engagement with incel forums has been associated with stronger endorsement of violent or harmful attitudes among some users (3). Networking is therefore best understood as a distinct amplifying context that may intensify either dimension, deepening distress for some users and consolidating grievance for others. Treating networking as an amplifying context rather than a third pathway is proposed here on the basis of this asymmetry, and was not tested as a moderation effect in the source study: it was roughly half as predictive as either dimension, and its content typically operates through them: forums supply validation for distress or reinforcement for grievance rather than an independent route to harm. Amplification is understood here to operate through increased exposure to ideological content, normalization of extreme views through group consensus, reduced contact with countervailing perspectives, and social reward for grievance expression.

This reciprocal, network-amplified process is the central psychological danger. Incel spaces may meet real psychological needs (belonging, explanation, identity, recognition) while satisfying them in a way that intensifies grievance and reduces agency (14).

Drawing together the evidence assembled in the preceding sections, the framework’s three provisional hypotheses can now be stated formally:

Hypothesis 1: Experiential vulnerability will show a comparatively stronger association with self-directed harm than with other-directed harmful attitudes.

Hypothesis 2: Ideological–dispositional risk will show a comparatively stronger association with other-directed harmful attitudes than with self-directed harm.

Hypothesis 3: Grievance-reinforcing online networking will amplify the association between experiential vulnerability and self-directed harm, and between ideological–dispositional risk and other-directed harmful attitudes, with effects varying according to whether online engagement primarily reinforces distress, fatalism, or misogynistic grievance. This amplification (moderation) effect was not tested in the source study and is advanced here as a novel prediction of the present framework.

These hypotheses require longitudinal, person-centered, and mediation/moderation designs.

## Self-directed and other-directed harm

The two-dimensional framework also clarifies harm, provided the relationship is stated carefully. Experiential vulnerability appears especially relevant to self-directed harm, including hopelessness, self-hatred, depression, and suicidal ideation. Ideological-dispositional risk appears especially relevant to other-directed harm, particularly when misogyny, entitlement, dehumanization, and revenge fantasies are present.

These are better understood as differences in relative emphasis rather than as a clean double dissociation. In the primary study underpinning this framework, both poor mental health and ideological adherence independently predicted other-directed harmful attitudes (displaced aggression, hostile sexism, and justification of violence), each roughly twice as predictive as online networking, even as the authors identified self-directed harm as the more prevalent risk (3). Experiential distress thus contributes to both outcomes; the two dimensions differ in emphasis, not in exclusive assignment. Some individuals may combine suicidal despair with homicidal ideation or symbolic revenge. This is why Hypotheses 1 and 2 are framed comparatively rather than exclusively: the evidence supports differences of emphasis, not categorical assignment.

The violence issue must be stated with care. High-profile attacks associated with incel or misogynistic grievance narratives demonstrate that incel ideology can be linked to serious violence (6). At the same time, available survey evidence suggests that most incel-identified individuals reject violence: in survey samples, roughly four in five do so (8, 22), and the most consistent signal across primary studies is that the dominant risk is directed at the self (3). This should be understood as a base-rate observation, not as reassurance that ideological content is harmless.

Risk assessment should distinguish online rhetoric from behavior, violent ideation from violent intent, and misogynistic belief from imminent threat (7, 23). Concerning indicators include explicit threats, weapon acquisition, leakage of intent, admiration for previous attackers, revenge fixation, recent humiliation, escalating dehumanization, suicidal intent combined with homicidal ideation, and immersion in online spaces that praise violence. Clinicians and safeguarding professionals should distinguish among violent ideation, active preparation, and imminent threat. Violent ideation warrants careful exploration; evidence of preparation warrants structured risk assessment and protective action; and imminent threat, particularly where a timeline and access to means are present, requires urgent intervention. These indicators derive primarily from general threat-assessment and grievance-fueled violence frameworks rather than from a validated incel-specific risk algorithm.

Policy and educational guidance often distinguishes personal, interpersonal, and societal harms (9). This is useful for practice, but it should be treated as safeguarding guidance rather than as primary psychological evidence. Personal harm includes self-harm and suicidality; interpersonal harm includes harassment or violence toward individuals; societal harm includes mass violence or ideologically framed attacks. This tripartite distinction is useful because it prevents an exclusive focus on terrorism while ignoring the more common problem of self-directed despair.

## Clinical implications

Clinical work should begin with differential assessment. The clinician should assess whether the person is experiencing unwanted celibacy, identifying with incel communities, endorsing incel ideology, expressing misogynistic grievance, or presenting acute risk. These are different clinical phenomena. In practice, endorsement of incel ideology is indicated by statements attributing romantic failure to immutable biological traits, female hypergamy, feminist corruption, or women’s alleged collective cruelty, whereas simple identification may involve use of the incel label without such explanatory commitments (7).

Assessment should include depression, anxiety, suicidality, self-harm, bullying history, trauma, social avoidance, rejection sensitivity, body image, perceived mate value, online activity, misogynistic beliefs, violent ideation, and access to means. The clinician should ask about incel-related online spaces directly but without ridicule. The goal is to understand what function the identity serves: belonging, explanation, emotional regulation, revenge fantasy, or protection from shame (14). Consistent with the dimensional framing outlined earlier, assessment should characterize internalizing distress and antagonistic or grievance-related features separately rather than treating incel identity as a diagnostic category.

Intervention should validate distress without validating ideology. A useful clinical stance is: “Your loneliness and despair are real; the conclusion that women are responsible for your suffering, or that your future is biologically fixed, does not follow.” This separates emotional validation from ideological agreement. Because incels tend to overestimate societal contempt for them (20, 21), gently testing this belief against evidence, rather than confirming or dismissing it, is a specific cognitive lever: examining the belief that “everyone hates me” may reduce the oppositional, victimized stance that sustains grievance, without minimizing genuine experiences of rejection. In practice this can take the form of behavioral experiments (for example, observing what actually happens when the client makes a small, low-stakes social overture), structured weighing of evidence for and against the belief, and perspective-taking exercises. These strategies are extrapolated from established treatments for depression, social anxiety, cognitive rigidity, and grievance-related thinking; their effectiveness specifically among incel-identified clients has not yet been evaluated.

Treatment may involve cognitive-behavioral work targeting hopelessness and deterministic beliefs, behavioral activation, graded social exposure, treatment of depression and anxiety, body-image work, social-skills practice, reduction of harmful online exposure, and development of non-misogynistic sources of belonging (7). Engagement is itself a challenge, since help-seeking is devalued within parts of the subculture; a non-judgmental, function-focused stance is therefore important. Where violent ideation, weapon access, or extremist identification is present, standard therapy alone is insufficient and should be integrated with safeguarding or threat-assessment procedures (23). For clients with social-communication difficulties, concrete and explicit skills work may be preferable to highly abstract approaches, although this recommendation is extrapolated from adjacent clinical literatures, and the pace and structure of therapy may need to be adapted accordingly. **Figure 2** summarizes this differentiated, domain-based response.

**Figure 2:**
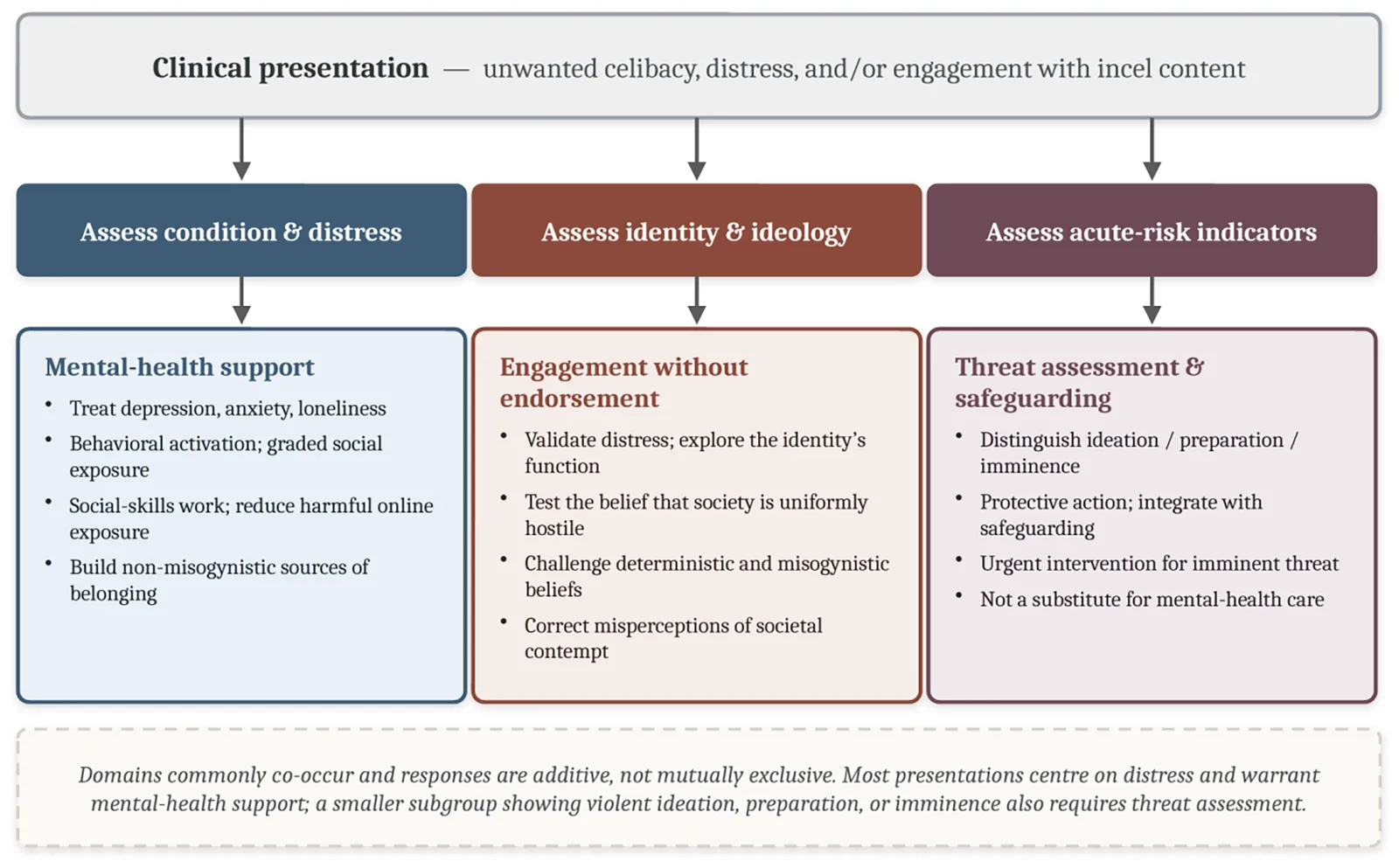
A differentiated, domain-based clinical response. Assessment spans three co-occurring domains (condition and distress, identity and ideology, and acute-risk indicators), each mapped to a corresponding response emphasis. Most presentations center on distress and warrant mental-health support; engagement with identity and ideology proceeds without endorsing the belief system; and threat assessment is reserved for the subgroup showing violent ideation, preparation, or imminence. Because the domains commonly co-occur, the responses are additive rather than mutually exclusive.

## Educational implications

For schools, colleges, and universities, the incel phenomenon should be addressed through mental-health support, online safety, respectful-relationships education, and misogyny prevention (9, 10). Educational responses should avoid sensationalism. Overexposure to attacker narratives can unintentionally glamorize violence or provide scripts for identification.

Staff should distinguish casual use of internet terminology from deeper ideological involvement. Some terms originating in incel spaces have migrated into broader online slang and do not necessarily indicate subcultural membership (9). Concern increases when terminology is accompanied by social withdrawal, hopelessness, misogynistic dehumanization, admiration for attackers, violent statements, or suicidal language.

Educational intervention should be proportionate. Curiosity or immature repetition of online terms may require conversation and education. Persistent despair may require mental-health referral. Threats, violent fixation, or explicit admiration for misogynistic attackers require safeguarding escalation (9, 23).

## Research limitations and future directions

The current evidence base has important limitations. Cross-sectional survey designs cannot establish the causal pathways this article describes. Forum analyses may overrepresent extreme users. Self-identified samples may exclude those who are ambivalent, hidden, or disengaged. Measures of incel identity are not yet standardized (1). More longitudinal, mixed-methods, and clinically informed research is needed.

Future research should examine exit pathways from incel communities and the turning points that precede them (10), protective factors, the distinction between self-directed and other-directed risk, the role of online networking and social-media algorithms, and the conditions under which loneliness becomes ideological grievance. Research should also extend beyond male, heterosexual incels to under-studied female incel communities (sometimes colloquially termed “femcels”), women who identify as involuntarily celibate, whose psychological profiles, experiences, and identity processes may differ in important ways. Reliable comparative data on the prevalence, patterns, and intensity of forum participation across male and female incel communities are currently lacking, which itself limits confident extension of the framework to femcels. They are excluded here not because they are unimportant, but because the gendered dynamics of mate value, entitlement, and grievance appear sufficiently different to require separate theoretical treatment rather than assimilation to a male-derived framework; this bounds the framework’s generalizability. Throughout, researchers should avoid amplifying extremist content unnecessarily. Scientific analysis must neither sanitize misogyny nor sensationalize socially isolated men.

Idiographic and case-based evidence would also help refine the framework. Systematic case studies, clinical case series, and analyses of individual trajectories, including those of people who entered and later left incel communities, could surface plausible mechanisms, temporal sequences, and boundary conditions that group-level cross-sectional data obscure: whether distress precedes ideological commitment or the reverse, which configurations precede self- versus other-directed outcomes, and what distinguishes those who disengage. Consistent with the aims stated here, such material should be used to generate and sharpen hypotheses rather than as confirmatory evidence of causality or prevalence, given the selection and recall limitations of case data.

## Discussion

Incel psychology is best understood through two interacting dimensions: experiential vulnerability and ideological–dispositional risk. Experiential vulnerability may be more closely associated with self-directed harm, whereas ideological–dispositional risk may be more closely associated with other-directed harm, although both contribute to both outcomes and online networking may amplify either. These hypotheses (Hypotheses 1–3) remain provisional because the underlying evidence is predominantly cross-sectional. The framework therefore supports differentiated, dimensional assessment rather than categorical labeling. Longitudinal and person-centered designs would provide the sharpest test: Hypotheses 1 and 2 would be disconfirmed if the two dimensions showed equivalent associations with self- and other-directed outcomes, and Hypothesis 3 if grievance-reinforcing engagement showed no amplifying effect on either association.

The framework’s value lies in separating phenomena that are often conflated. Unwanted celibacy, incel self-identification, misogynistic ideology, self-directed harm, and other-directed harmful attitudes are related but non-equivalent constructs. Treating them as separable allows future studies to test whether different configurations of mental-health vulnerability, ideological commitment, and online reinforcement predict different outcomes.

The most defensible response is differentiated. Involuntary celibacy is not pathology. Incel identity is not automatically violence risk. Incel ideology, however, may be psychologically and socially harmful, particularly when it combines fatalism, misogyny, self-hatred, and revenge. Effective intervention requires validating suffering, restoring agency, challenging deterministic and misogynistic beliefs, examining misperceptions of societal contempt, reducing harmful online reinforcement, and applying careful risk assessment when self-harm or violence indicators are present.

## Data Availability

The original contributions presented in the study are included in the article/supplementary material. Further inquiries can be directed to the corresponding author.
